# Artificial Balance: Restoration of the Vestibulo-Ocular Reflex in Humans with a Prototype Vestibular Neuroprosthesis

**DOI:** 10.3389/fneur.2014.00066

**Published:** 2014-04-29

**Authors:** Angelica Perez Fornos, Nils Guinand, Raymond van de Berg, Robert Stokroos, Silvestro Micera, Herman Kingma, Marco Pelizzone, Jean-Philippe Guyot

**Affiliations:** ^1^Service of Otorhinolaryngology and Head and Neck Surgery, Department of Clinical Neurosciences, Geneva University Hospitals, Geneva, Switzerland; ^2^Division of Balance Disorders, Department of Otorhinolaryngology and Head and Neck Surgery, Faculty of Health Medicine and Life Sciences, School for Mental Health and Neuroscience, Maastricht University Medical Center, Maastricht, Netherlands; ^3^Translational Neural Engineering Laboratory, Center for Neuroprosthetics, Institute of Bioengineering, École Polytechnique Fédérale de Lausanne, Lausanne, Switzerland; ^4^The BioRobotics Institute, Scuola Superiore Sant’Anna, Pisa, Italy

**Keywords:** vestibular implant, balance, sensory neuroprostheses, rehabilitation, vestibulo-ocular reflex

## Abstract

The vestibular system plays a crucial role in the multisensory control of balance. When vestibular function is lost, essential tasks such as postural control, gaze stabilization, and spatial orientation are limited and the quality of life of patients is significantly impaired. Currently, there is no effective treatment for bilateral vestibular deficits. Research efforts both in animals and humans during the last decade set a solid background to the concept of using electrical stimulation to restore vestibular function. Still, the potential clinical benefit of a vestibular neuroprosthesis has to be demonstrated to pave the way for a translation into clinical trials. An important parameter for the assessment of vestibular function is the vestibulo-ocular reflex (VOR), the primary mechanism responsible for maintaining the perception of a stable visual environment while moving. Here we show that the VOR can be artificially restored in humans using motion-controlled, amplitude modulated electrical stimulation of the ampullary branches of the vestibular nerve. Three patients received a vestibular neuroprosthesis prototype, consisting of a modified cochlear implant providing vestibular electrodes. Significantly higher VOR responses were observed when the prototype was turned ON. Furthermore, VOR responses increased significantly as the intensity of the stimulation increased, reaching on average 79% of those measured in healthy volunteers in the same experimental conditions. These results constitute a fundamental milestone and allow us to envision for the first time clinically useful rehabilitation of patients with bilateral vestibular loss.

## Introduction

Balance can be considered as the sixth human sense. It is the result of the synergistic processing of multisensory information that allows for the unconscious automatization of essential tasks such as postural control, gaze stabilization, and spatial orientation. The peripheral vestibular system is part of the inner ear and it is composed of multi-dimensional motion sensors, located in the semicircular canals and in the otolithic organs. These end organs are connected to the branches of the vestibular nerve and provide information crucial to balance tasks, and any limitation in their function can significantly affect balance control. This is particularly true in the case of a bilateral loss of vestibular function (BVL). Recent studies have clearly demonstrated that there are significant functional consequences to this deficit (1, 2) and that these have a significant adverse impact on the quality of life of affected patients (3). Currently, there is no evidence of an effective treatment for patients with BVL. Despite intensive balance retraining, most patients show no long-term improvement of their symptoms or recovery of their vestibular function (4).

The idea of electrically stimulating the vestibular system in an attempt to rehabilitate patients with a BVL is based on a concept very similar to that of cochlear implants, which are very successful for rehabilitating patients with profound hearing loss (5). Extensive animal studies provided the first evidence supporting the feasibility of the idea. Already in 1963, Cohen and Suzuki showed that electrical stimulation of an ampullary branch of the vestibular nerve induces a nystagmic response predominantly in the plane of the stimulated branch (6). Several decades later, Merfeld’s team at the Jenks Vestibular Physiology Laboratory (Massachusetts Eye and Ear Infirmary, Boston, MA, USA) provided the first demonstration of the ability to generate smooth eye movements in response to bilateral (7, 8) and unilateral (9, 10) electrical stimulation of the ampullary branches of the vestibular nerve. The possibility of coupling motion sensors to the head and using this information to modulate the electrical stimulation delivered to the vestibular system was also systematically investigated and verified in animal models by the same authors (8, 9, 11) and also by Della Santina’s team at the Vestibular NeuroEngineering Laboratory [Johns Hopkins School of Medicine, Baltimore, MA, USA; Ref. (12–15)].

Although these studies have achieved important steps in demonstrating the feasibility of the concept, it is very difficult to assess the true functional benefits that humans could derive from such a system via animal studies. First, in all animals the BVL was induced chemically or mechanically (canal plugging), which might differ significantly from the natural BVL pathophysiology. Indeed, BVL can be of various etiologies, of different durations, and thus with different levels of neurosensory involvement (16). Second, for obvious reasons it is difficult to assess the true functional benefits of a vestibular neuroprosthesis in animals. These limitations highlight the need of human research to verify the potential clinical benefit of this approach.

To our knowledge, there are only two groups in the world investigating the use of electrical stimulation of the vestibular system in human patients: our group and the team lead by Rubinstein at the University of Washington (Washington, DC, USA). However, their concept is fundamentally different from ours and from the one developed by Merfeld’s and Della Santina’s teams. Instead, their first approach was to develop a “vestibular pacemaker,” not intended to code motion (17). This device was directed at patients with Menière disease, in the hope to control the repeated, transient episodes of vertigo associated with the disease, but should preserve vestibular function, which is close to normal in this group of patients. They have also conducted preliminary animal studies confirming the possibility to electrically elicit eye movements (and thus effectively activate the vestibular system) with their device, and suggested that it was possible to preserve hearing and pre-existing vestibular function after implantation (18). Unfortunately, this was not verified in humans and both the auditory and vestibular function of implanted patients deteriorated considerably (17, 19). Recently, this group shifted focus to a motion coding device, as mentioned in a recent publication exploring the possibility to induce body sway upon computer-controlled electrical stimulation (19).

Our research consortium has fulfilled several important prerequisites for the development of a system allowing for the chronic stimulation of the peripheral vestibular system in human patients (20). Special surgical techniques have been developed (21–24) and the feasibility of electrical stimulation of the three different ampullary branches of the vestibular nerve has been demonstrated both in acute (25, 26) and chronic settings (27). Yet, a fundamental milestone to establish the validity of the approach should be accomplished: to investigate whether electrical stimulation of the ampullary branches of the vestibular nerve could provide a means to effectively rehabilitate patients with BVL.

In this context, the vestibulo-ocular reflex (VOR) is of particular interest. It is one of the gold standards in vestibular testing since it can be easily quantified (gain, phase, and axis) and since it provides objective evidence of the ability to restore gaze stabilization mechanisms in patients with BVL in specific conditions. The VOR is a reflex eye movement that allows maintaining stable gaze on the object of interest by producing an eye movement in the direction opposite to the head movement. When bilateral vestibular function is lost, the mechanisms generating these compensatory eye movements for image stabilization are inadequate. A direct functional consequence of this lack of image stabilization is an abnormal decrease of dynamic visual acuity, which makes face recognition and sign reading difficult while walking (1, 2). Therefore, as a first step in the evaluation of the rehabilitation prospects of a vestibular neuroprosthesis, we decided to investigate whether the artificial restoration of the VOR is possible via electrical stimulation of an ampullary branch of the vestibular nerve, in humans suffering from BVL of different etiologies and different disease durations. For practical reasons, we focused on the stimulation of the lateral ampullary nerve (LAN), which generates in theory a one-dimensional horizontal angular VOR. Indeed, it is much easier to deliver horizontal than vertical whole-body rotations.

## Materials and Methods

We investigated whether it is possible to artificially restore the VOR in patients with BVL. We hypothesized that this could be achieved using motion-controlled, amplitude modulated electrical stimulation. Tests were performed without any electrical stimulation (system OFF) and upon electrical stimulation of the LAN (system ON). In the system ON condition, the amplitude of the electrical stimulation was modulated via the motion signal captured by an inertial sensor (gyroscope).

### Patients

Three patients with BVL (see Table 1) participated in the experiments. Patients were recruited at the Service of Otorhinolaryngology and Head and Neck Surgery at the Geneva University Hospitals and at the Division of Balance Disorders at the Maastricht University Medical Center. They fulfilled the following inclusion criteria: (1) mean peak slow phase velocity of 5°/s or less in bilateral bithermal (30 and 44°C) caloric irrigations with water, (2) pathologic head-impulse test for horizontal and vertical semicircular canals, and (3) a VOR gain of <0.25 on rotatory chair tests. In addition, since there is a non-negligible risk of inducing a profound hearing loss with the implantation surgery (5, 17, 19, 20), we chose patients who were also profoundly deaf in the implanted ear (i.e., and thus could benefit from a cochlear implant).

**Table 1:** **Demographics and stimulation details of the three implanted patients**.

	Sex	Age (implantation)	BVL etiology	Deafness	Implanted ear	Vestibular threshold (μA)	UCL (μA)	Steady-state stimulation amplitude (μA)
BVL1	Female	58	Meningitis	Unilateral	Right	100	225	160
BVL2	Male	66	DFNA9	Bilateral	Left	145	345	245
BVL3	Female	67	Trauma	Bilateral	Left	175	425	300

*UCL, upper comfortable level*.

### Device

**Figure 1:**
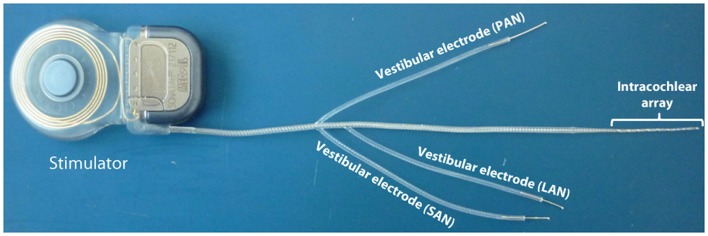
**Vestibular implant prototype**. An out-of-the-shelf cochlear implant (SONATA; MED-EL, Innsbruck, Austria) was modified in order to provide electrodes for stimulating vestibular structures. Three electrodes were taken out from the standard intracochlear array. Each of these “vestibular” electrodes was located on the distal tip of separate leads to allow implantation in the posterior, superior, and lateral ampullae.

Patients received a vestibular neuroprosthesis prototype consisting of modified cochlear implant (MED-EL, Innsbruck, Austria). This device, in addition to the cochlear electrode array, provided extracochlear electrodes, which were implanted in the vicinity of the ampullary branches of the vestibular nerve (see Figure 1).

Implantation was performed using an intralabyrinthine surgical approach. Briefly, this procedure consisted in a regular cochlear implant retroauricular approach with extension of the mastoidectomy. Semicircular canals were blue lined adjacent to their ampullary ends. Millimetric fenestrations adjacent to each ampullary end were made to allow selective intralabyrinthine access. This surgical approach has been presented in detail in a previous publication (21).

### Study design

This study consisted of controlled laboratory experiments during which subjects were submitted to horizontal whole-body rotations (i.e., around the vertical axis) in complete darkness (without head stabilization). Rotation velocities had a sinusoidal profile with 30°/s peak amplitude, based on the typical motion profile of human locomotion (28, 29). Five different rotation frequencies (0.1, 0.25, 0.5, 1, and 2 Hz) were tested. Rotations were realized with a custom-modified, velocity controlled rotatory chair (Nystagliner Pro; Erich Jaeger Gmbh).

### Electrical stimulation

When the prototype device was activated (system ON), electrical stimulation was exclusively delivered to the LAN (the vestibular nerve branch naturally responding to rotations around the vertical axis). Stimulation consisted of trains of charge-balanced, biphasic pulses (200 μs/phase) presented at 400 pulses/s. As described previously, the activation of unilateral electrical stimulation of the vestibular system requires a two-step process (10, 27). Briefly, the patients first received constant amplitude electrical stimulation on their LAN for 30 min to ensure that all vestibular symptoms (e.g., vertigo, nystagmic responses) vanished. This steady-state, constant amplitude stimulation stage served to artificially restore a baseline or “spontaneous” firing rate in their deafferented nerve. Then, only when they were “adapted” to the steady-state stimulation, the amplitude of the electrical stimulation could be increased (up-modulated) for generating eye movements in one direction and decreased (down-modulated) for generating eye movements in the opposite direction. In our experiments, motion information was captured by a three-axis gyroscope (LYPR540AH; ST Microelectronics; Geneva, Switzerland) and its signal was used to up- and down-modulate the amplitude of the train of pulses delivered via the vestibular electrode.

The current values of the steady-state stimulation for each patient were chosen to correspond approximately to the middle of the previously measured dynamic range (i.e., from the vestibular activation threshold to the upper comfortable level) of each patient (see Table 1). To evaluate the effect of the intensity of the stimulation on the VOR response, two modulation strengths (i.e., electrical gains of the gyroscope) were tested per patient. These modulation strengths were equivalent to modulation depths covering 50 and 75% of each patient’s measured dynamic range at the 30°/s peak-velocity of sinusoidal rotations.

### Eye movements recording and analysis

During the experiments, eye-in-head angular position (30) was recorded with a 2D video-oculography system incorporating six DOF head motion sensors [EyeSeeCam VOG; Munich, Germany; Ref. (31)]. This choice was motivated because of its light-weight design (e.g., minimizing artifacts due to goggle slippage), its high frame rate (approximately 220 frames/s), and because it is minimally invasive and thus causes minimum discomfort to the subject. However, it has the disadvantage that it only records bi-dimensional eye movements (no torsion). We decided to use the EyeSeeCam system and to not take eye torsion into account since did not observe any visible (to the naked eye) torsional components during preceding LAN stimulation sessions. Therefore, even if minimal torsion existed, it would have had little impact on VOR responses.

**Figure 2:**
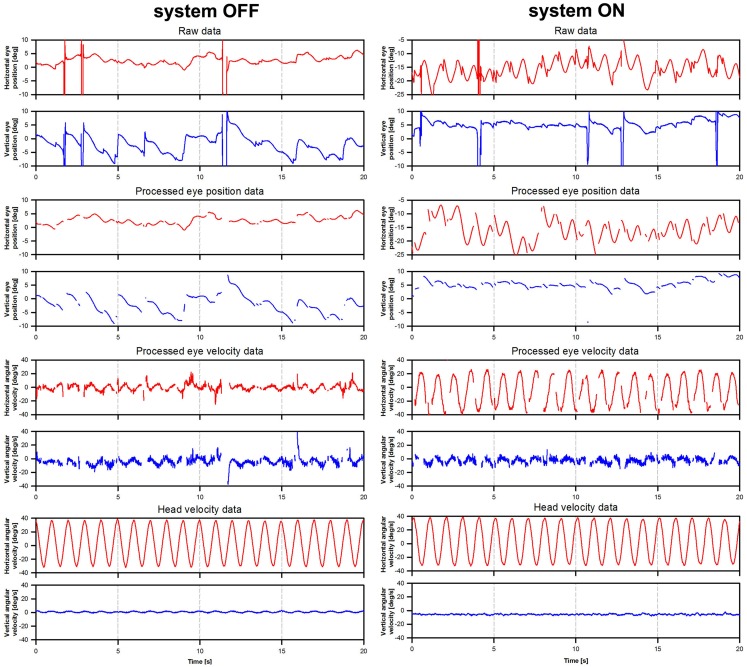
**Illustration of eye movement data processing**. The figure presents eye movement data tracings for patient BVL1, gathered during the rotation experiments at a frequency of 1 Hz (modulation strength corresponding to 50% of the patient’s dynamic range). The panels on the left show data acquired during the system OFF experiments. The panels on the right show data gathered during the system ON experiments. Three steps are illustrated: raw eye position (e.g., before any processing was performed), processed eye position data (e.g., eye position data after blinks and quick eye movements >1000°/s^2^ were removed), and processed eye velocity data (e.g., obtained from the differentiation of the processed eye position data).

A segment of 21 rotation cycles was analyzed for each experimental trial. Eye velocity and acceleration were obtained via the first and second derivatives of eye position. Blinks and quick eye movements (e.g., saccades and nystagmus quick-phases) were detected as segments where eye acceleration was >1000°/s^2^. These segments were removed and were not replaced by interpolated values. Examples of raw eye movement data and subsequent processing are presented in Figure 2.

We performed a Fourier analysis on the angular eye velocity data to verify the assumption that the response frequency was the same as the stimulus (i.e., rotation) frequency. The proportion of the signal energy accounted by the stimulus frequency was >90% in most system ON conditions. This proportion was lower in the cases where the VOR response was absent.

Vestibulo-ocular reflex response was analyzed on the basis of eye and head velocity data, using a cycle-by-cycle analysis. For each cycle, best-fit frequency-fixed sinusoids to horizontal (yaw) eye and head velocity data were computed. In addition, since a mandatory characteristic of compensatory eye movements is that they should be in counter-phase to head movements (ideally a phase-shift of 180°), we imposed an additional mathematical constraint to calculate gain only on the basis of compensatory eye movements. We used the magnitude and also the phase-shift results of the best-fit analysis to compute the phase-locked VOR gain. Briefly, best-fit responses are characterized by a magnitude (amplitude of the sinusoidal best-fit) and a phase. The raw VOR gain was first computed as the ratio of the best-fit eye and head velocity magnitudes. The phase-shift was computed as the difference in phase between best-fit eye and head responses. Then, the phase-locked VOR gain was computed using vector projection calculations on the “ideal” VOR vector. Briefly, a vector projection can be defined as the scalar projection of vector *A* on vector *B*, defined by the following equation:

$$A_{||B}=|A|\cos\theta \frac{B}{\left|B\right|}$$

where *A*_||_*_B_* is the resulting vector projection (the phase-locked VOR gain), |*A*| is the magnitude of the original vector (the raw VOR gain), and *B* represents the ideal VOR vector (with a magnitude |*B*| of 1 and phase-shift of 180°). θ is the angle between the raw VOR and “ideal” VOR vectors.

This calculation would result, for example, in an unaltered gain for eye movements having the “ideal” 180° phase-shift with respect to head movements, and in negative gains for eye movements that were in phase (e.g., a phase-shift of 0°) with head movements.

### Statistics

Individual patient results were not normally distributed. Therefore, results are presented as median (25th–75th percentile) values and non-parametric tests were used for statistical analyses. Group (pooled) results followed a normal distribution. Therefore, results are presented as mean values (±standard error of the mean, SEM) and parametric tests were used for statistical analyses. Results of the normality (Shapiro–Wilk) and equal-variance tests are presented in each case.

### Ethical considerations

Experiments were designed and conducted in accordance with the Declaration of Helsinki. Local ethical committees of the Geneva University Hospitals (NAC 11-080) and of the Maastricht University Medical Center (NL36777.068.11/METC 11-2-031) gave their approval to this experimental protocol.

### Control measures with healthy volunteers

Five healthy volunteers, familiar with the purpose of the study, were included as a control group (three male and two female; mean age 43 years; age range 35–59 years) and signed appropriate consent forms. They had no prior vestibular symptoms and had normal head-impulse test findings in the three semicircular canal planes. Results for this control group are presented in Figure S1 in Supplementary Material.

## Results

### VOR response as a function of rotation frequency

**Figure 3:**
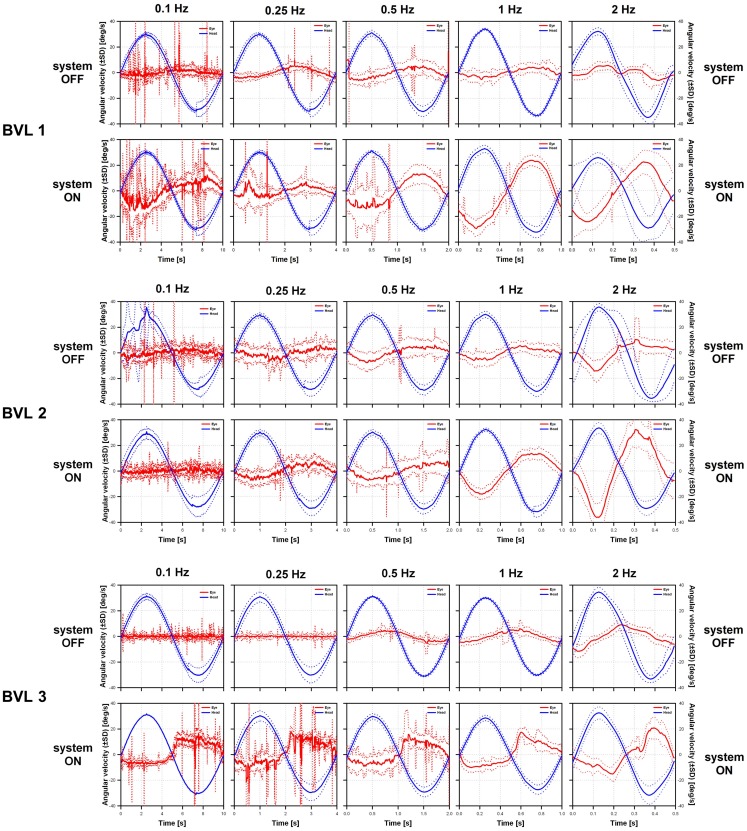
**Vestibulo-ocular reflex responses of the three implanted patients to 30°/s peak-velocity sinusoidal rotations around the vertical axis in complete darkness at frequencies of 0.1, 0.25, 0.5, 1, and 2 Hz (columns)**. For each patient, the panels on the upper row show data gathered in the system OFF condition. The panels on the lower row show data gathered in the system ON condition. Solid lines represent the average cycle plots (±standard deviation, SD shown in dotted lines) of the horizontal angular velocity of the eye (red lines) and the head (blue lines). Note that at the lower frequencies eye movement recordings were polluted by random artifacts mainly due to the long duration of cycles at these frequencies (e.g., 10 s for 0.1 Hz rotations).

**Figure 4:**
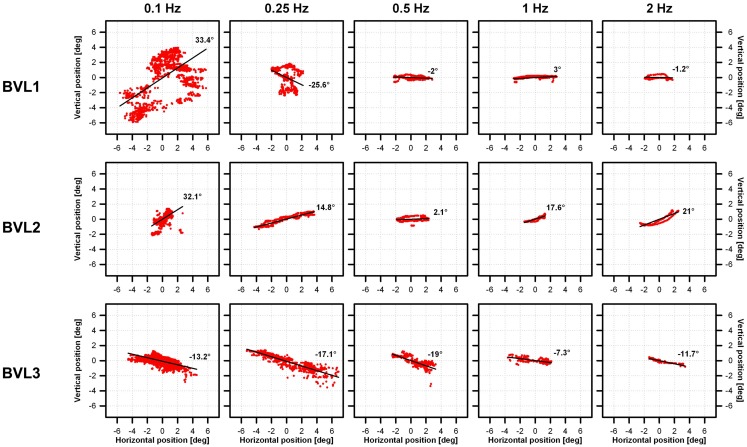
**Vestibulo-ocular reflex response axes of the three implanted patients to 30°/s peak-velocity sinusoidal rotations around the vertical axis in complete darkness at frequencies of 0.1, 0.25, 0.5, 1, and 2 Hz (columns)**. Each individual panel shows average vertical versus horizontal eye position (red dots) gathered for each patient (rows) in the system ON condition (modulation strength corresponding to 50% of the dynamic range), at the different rotation frequencies tested (columns). The best linear fits to the data were calculated to estimate the axis of eye movements (angle with respect to the horizontal). Important eye dispersion was observed for the lower frequencies and the linear fits were not representative of the direction of eye movements. This was expected since the VOR response at these frequencies was almost absent and in these cases eye movement patterns were mainly random due to the long duration of cycles at these frequencies (e.g., 10 s for 0.1 Hz rotations). Otherwise eye movements were predominantly horizontal (maximum deviations of 2°, 21°, and 19° for BVL1, BVL2, and BVL3, respectively), and the axis remained almost the same despite the rotation frequency tested.

Examples of VOR responses of the tested patients to 30°/s peak-velocity sinusoidal rotations in darkness, at five different frequencies (0.1, 0.25, 0.5, 1, and 2 Hz) are presented in Figure 3. In the “ideal” VOR response, the time-course of eye movements should mirror that of head movements, following an inverted compensatory pattern. However, as a consequence of the BVL, compensatory eye movements (red lines in Figure 3) were practically absent for all three patients in the system OFF conditions. In contrast, a clear VOR response was observed in the system ON condition, especially at the highest rotation frequencies (1 and 2 Hz). In these conditions, the time-course of eye movements begins to mirror that of head movements (blue lines in Figure 3), replicating the typical characteristics of the “ideal” VOR of healthy subjects. Furthermore, the axis of the electrically evoked VOR was consistent with horizontal motion and remained practically unchanged for the five rotation frequencies tested (see Figure 4).

**Figure 5:**
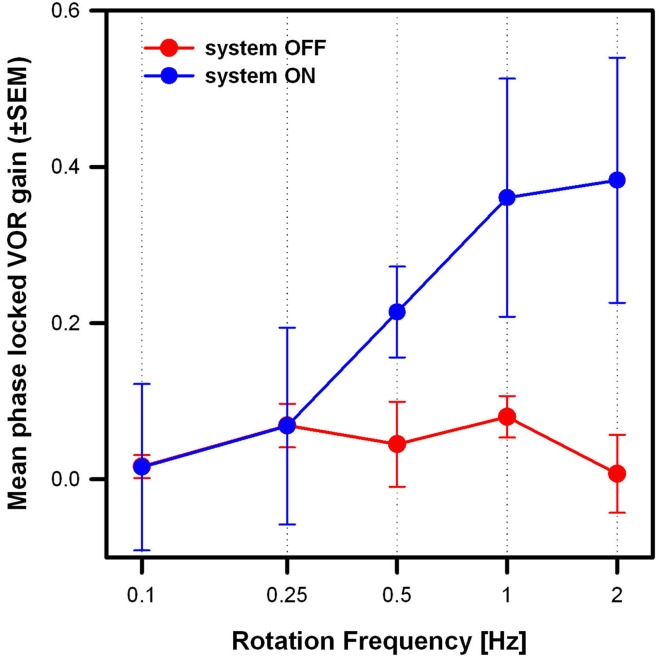
**Mean phase-locked VOR gain (±SEM) versus rotation frequency of the three implanted patients**. Two stimulation conditions are compared: system OFF (red plot) and system ON (blue plot; modulation strength corresponding to 50% of each patients dynamic range).

Figure 5 compares the mean results of the three patients in the system OFF and system ON conditions (modulation strength corresponding to 50% of each patient’s dynamic range), at the five rotation frequencies tested. Mean phase-locked VOR gain increased significantly from the system OFF to the system ON condition [two-way repeated measures analysis of variance (ANOVA); *F*_(1,15)_ = 19.63, *p* = 0.04; Shapiro–Wilk *p* = 0.067; equal-variance *p* = 0.87]. In the system OFF condition, the gain was low (<0.1) at all rotation frequencies. In the system ON condition, the gain was also low for rotation frequencies of 0.1 and 0.25 Hz, and increased monotonically for rotation frequencies of 0.5 Hz and above. Pairwise *post hoc* comparisons (Tukey Test) indicated significant differences between the system OFF and system ON conditions at 1 Hz (*p* = 0.02) and at 2 Hz (*p* = 0.01).

Individual patient results are presented in Table 2. Results of Friedman repeated measures analysis of variance (FRMAV) tests indicated a statistical effect of stimulation condition for all patients [BVL1: χ^2^_(9, *N*=21_) = 81.63, *p* < 0.001, Shapiro–Wilk *p* < 0.05; BVL2: χ^2^_(9, *N*=21)_ = 71.35, *p* < 0.001, Shapiro–Wilk *p* < 0.05; BVL3: χ^2^_(9, *N*=21)_ = 83.08, *p* < 0.001, Shapiro–Wilk *p* < 0.05]. *Post hoc* comparisons (Tukey Test) indicated statistically significant gain increases in the system ON conditions at 1 and 2 Hz for patients BVL1 and BVL2. In the case of patient BVL3, the gain increase in the system ON condition was significant (*p* < 0.05) at all rotation frequencies.

**Table 2:** **Phase-locked VOR gain at the five rotation frequencies tested for the three implanted patients**.

	Rotation frequency									
	0.1 Hz		0.25 Hz		0.5 Hz		1 Hz		2 Hz	
Patient	OFF	ON	OFF	ON	OFF	ON	OFF	ON	OFF	ON
**BVL1**	0.04	−0.06	0.11	−0.16	0.17	0.35	0.16	0.66	0.13	0.86
	(−0.09–0.12)	(−0.32–0.22)	(0.07–0.15)	(−0.25–0.12)	(0.08–0.23)	(−0.51–0.54)	(0.04–0.21)	(0.61–0.76)	(−0.11–0.18)	(0.57–0.99)
**BVL2**	−0.01	−0.05	0.10	0.19	0.18	0.20	0.12	0.35	0.12	0.27
	(−0.16–0.14)	(−0.13–0.13)	(−0.05–0.24)	(0.04–0.25)	(0.01–0.30)	(0.11–0.33)	(−0.10–0.20)	(0.30–0.39)	(0.04–0.19)	(0.17–0.41)
**BVL3**	0.04	0.27	0.01	0.14	−0.06	0.16	0.06	0.26	0.00	0.19
	(−0.04–0.07)	(0.08–0.30)	(−0.03–0.05)	(0.00–0.27)	(−0.10–0.02)	(0.11–0.19)	(0.00–0.10)	(0.21–0.31)	(−0.08–0.08)	(0.14–0.23)
**Mean**	0.02	0.05	0.07	0.06	0.10	0.24	0.11	0.43	0.08	0.44
**(SEM)**	(0.02)	(0.11)	(0.03)	(0.11)	(0.08)	(0.06)	(0.03)	(0.12)	(0.04)	(0.21)

*Two stimulation conditions are compared: system OFF and system ON (modulation strength corresponding to 50% of each patient’s dynamic range)*.

*Values given per patient are median (calculated over 21 cycles); 25th–75th range given in parentheses*.

*SEM, standard error of the mean*.

### VOR response as a function of stimulation intensity

The previous results demonstrated significant group and individual improvements in the phase-locked VOR gain when the system was turned ON. However, for patients BVL2 and BVL3, the increase was moderate (see Table 2). The next experiment was designed to investigate whether those moderate gains could be increased by augmenting the intensity of stimulation. For this experiment, patients were again submitted to 30°/s peak-velocity sinusoidal rotations in darkness (around the vertical axis) at 1 Hz in the system ON condition, but the modulation strength was increased to 75% of each patient’s dynamic range.

**Figure 6:**
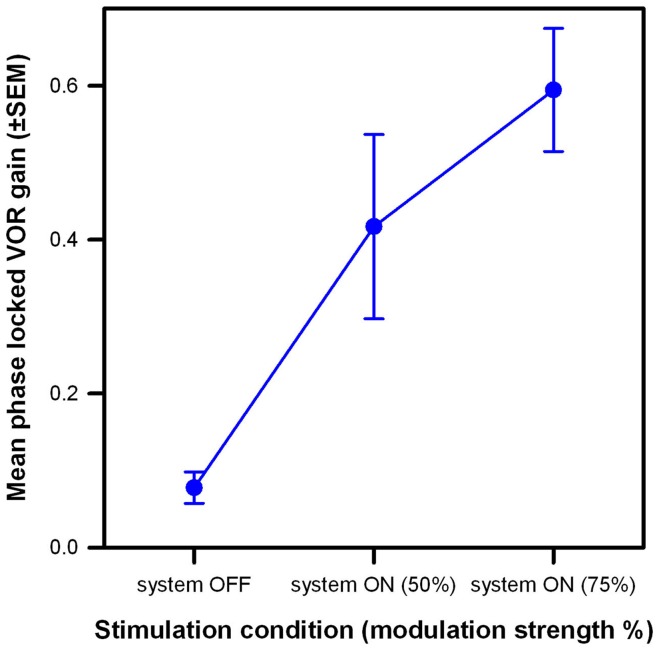
**Mean phase-locked VOR gain (±SEM) versus stimulation condition of the three implanted patients**. Three stimulation conditions are compared: system OFF and system ON with modulation strengths corresponding to 50 and 75% of each patient’s dynamic range. Rotations had a 30°/s peak-velocity sinusoidal profile (around the vertical axis) at 1 Hz.

The results of this experiment are summarized in Figure 6. The low phase-locked VOR gain observed in the system OFF condition (mean 0.11, SEM 0.03) increased monotonically as stimulation intensity increased, reaching mean values of 0.43 (SEM 0.12) and 0.59 (SEM 0.08) at modulation strengths corresponding to 50 and 75% of the patients’ dynamic range, respectively. A one-way ANOVA was conducted to confirm this effect. There was a statistically significant difference in the mean phase-locked VOR gain of the three patients [*F*_(2,8)_ = 15.3, *p* = 0.01; Shapiro–Wilk *p* = 0.53; equal-variance *p* = 0.65]. *Post hoc* comparisons using the Tukey Test indicated that the mean gain was significantly higher (*p* < 0.05) in both system ON conditions than in the system OFF condition. The difference between both system ON conditions was not statistically significant.

**Figure 7:**
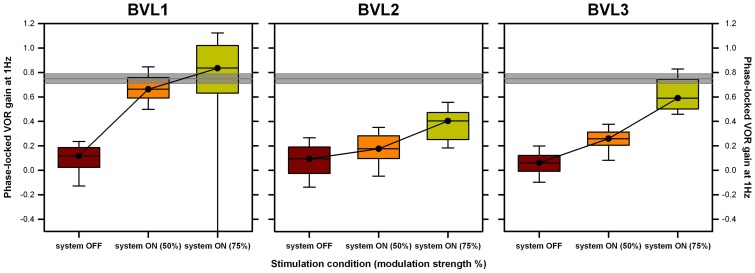
**Individual VOR performance recorded during the rotation experiments at 1 Hz**. Each panel presents data gathered for one patient. Three conditions are compared: system OFF and system ON at two different modulation strengths (corresponding to 50 and 75% of each patient’s dynamic range, in parenthesis). Data were analyzed on a cycle-by-cycle basis. Box plots indicate median values, 25th and 75th percentile values (colored box) as well as 10th and 90th percentile values (error bars). The gray bar represents the mean ± SEM of the VOR gain measured in a group of five healthy subjects in the same experimental conditions.

Individual patient results follow the same trend as pooled group results (see Figure 7). Results of a FRMAV test indicated that there was a statistically significant difference across the system OFF and the system ON conditions [BVL1: χ^2^_(2, *N*=21)_ = 19.81, *p* < 0.001, Shapiro–Wilk *p* < 0.05; BVL2: χ^2^_(2, *N*=21)_ = 32.09, *p* < 0.001, Shapiro–Wilk *p* = 0.06, equal-variance *p* < 0.05; BVL3: χ^2^_(2, *N*=21)_ = 40.09, *p* < 0.001, Shapiro–Wilk *p* < 0.05]. *Post hoc* pairwise comparisons (Tukey Test) showed a significant increase (*p* < 0.05) in phase-locked VOR gains between the system OFF condition and both system ON conditions for all patients (BVL1: median 0.66, 25th–75th percentiles 0.61–0.76 at 50% and median 0.91, 25th–75th percentiles 0.79–1.08 at 75%; BVL2: median 0.35, 25th–75th percentiles 0.30–0.39 at 50% and median 0.43, 25th–75th percentiles 0.26–0.55 at 75%; BVL3: median 0.26, 25th–75th percentiles 0.21–0.31 at 50% and median 0.59, 25th–75th percentiles 0.50–0.74 at 75%). The difference between the phase-locked VOR gains obtained in both system ON conditions (different modulation strengths) was statistically significant (*p* < 0.05) only for BVL3.

## Discussion

The present study was designed to investigate whether it is possible to evoke an artificial motion modulated VOR response via electrical stimulation of the LAN of the vestibular nerve in humans with a BVL. As expected, our implanted patients showed low (<0.1) phase-locked VOR gains in the system OFF condition. These increased significantly in the system ON conditions, and as modulation strength (i.e., intensity of the stimulation) increased, reaching 57–121% (mean 79%) of the median phase-locked VOR gain measured for a group of five healthy subjects in the same experimental conditions (gray bars in Figure 6; mean 0.75, SEM 0.04; see Figure S1 in Supplementary Material) and approaching the VOR performance reported for a group of healthy volunteers during walking [median 96, 25th–75th percentiles 0.89–1.03; Ref. (28)]. This artificial restoration of the VOR in our group of implanted patients can be therefore considered as substantial functional recovery. Furthermore, this was possible for patients with substantially different deficit durations (from >50 years in the case of BVL1 to 1 year in the case of BVL3) and with different BVL etiologies.

It is interesting to point out that these results were achieved without any parametric optimizations of the electrical stimulation paradigm. For example, as a first approximation we assumed a linear relationship between electrical stimulation and the evoked eye movement response. Measurements showed that the electrically evoked VOR was slightly asymmetrical (see Figure 3; Figure S2 in Supplementary Material), suggesting a non-linear relationship between stimulation current and velocity of the evoked eye movement. This is not surprising: the non-linear nature of the relationship between the electrical stimulus and the neural response is well-known and compensated in other sensory neuroprosthetic systems (e.g., cochlear and retinal implants). Parametric optimizations of the stimulation paradigm could therefore be implemented to improve this, for example, employing a non-linear (e.g., logarithmic) transfer function between the input (i.e., detected head angular velocity) and the output (i.e., electrical currents delivered via the stimulated electrodes). Conversely, results of some animal experiments suggest that the vestibular system itself is able to adapt to a “suboptimal” stimulus and improve the symmetry of the response (as well as other parameters such as VOR gain) with time (9). Our previous studies suggest that vestibular adaptation processes are much faster in humans than in animals (27). Therefore, the characteristics of the artificially evoked VOR might improve rapidly as patients get used to wearing the vestibular neuroprosthesis. It will be thus of high interest to repeat all functional tests after extended periods of active electrical stimulation.

We also observed better artificial VOR responses at higher rotation frequencies. At rotation frequencies of 0.1 and 0.25 Hz, the artificially evoked VOR was practically absent. The VOR response started to grow at 0.5 Hz, reaching maximum performance at rotation frequencies of 1 and 2 Hz. This resembles well-known and documented dynamic characteristics of the normal VOR (32, 33). While for low rotation frequencies (<0.5 Hz), the normal VOR gain is less than unity and variable, in the range of 0.5–2 Hz the gain of the normal VOR increases and is close to unity. These dynamic characteristics of the VOR probably reflect the functional demands of gaze stabilization mechanisms (34, 35), optimizing visual performance in certain situations (e.g., simultaneous eye and head movement to follow a moving target). On the contrary, VOR gain is greater at higher frequencies >0.5 Hz, where it can no longer be suppressed by vision. In this higher frequency range, its role in stabilizing gaze becomes therefore predominant. Important every-day activities such as walking induce head movements predominantly in the 1–2 Hz frequency range (28, 29, 32, 36–38). Therefore, our results demonstrate that it is possible to restore an artificial VOR in a critical frequency range, and allow us to envision useful restoration of gaze stabilization mechanisms in patients with a BVL during important every-day activities.

In order to provide a more accurate evaluation of the functional rehabilitation prospects, future efforts will concentrate on extending these results to other essential tasks such as postural control and to objectively evaluate the benefit of artificially restoring the VOR during every-day activities. In addition, another important aspect worth mentioning is that our experimental paradigm allows direct selective stimulation of three different vestibular end organs. In other words, our setup allows for the de-coupling of vestibular information from whole-body information, which is normally impossible to achieve in “natural” conditions. We hope that this particular experimental paradigm will help us achieve a better understanding of the multisensory processing of motion.

Finally, it is important to point out the fact that in our experiments only the LAN electrode was activated. In real life all electrodes, cochlear and vestibular, will be activated. Our experimental choice was motivated on the fact that, as a first step, we wanted to separately evaluate a one-dimensional angular VOR. In addition, we wanted to avoid any possible bias coming from the other “artificial” sensory modality. Interestingly, there have been some reports of improved balance in cochlear implant wearers when their implant is turned on [see e.g., Ref. (39)]. That being said, we do not expect significant interactions between cochlear and vestibular electrodes because of the long distance between them, and because of interleaved stimulation strategies (40, 41). However, in the future it will be interesting to compare performance in both situations in order to investigate how both cochlear and vestibular systems interact with each other.

In conclusion, the results achieved with these three patients constitute a fundamental milestone, demonstrating that the artificial restoration of the VOR is possible via electrical stimulation of the LAN branch of the vestibular nerve in humans. In fact, these results provide the first objective evidence that gaze stabilization mechanisms in patients with a BVL can be restored using electrical stimulation, in conditions replicating the typical motion profile of important every-day tasks such as human locomotion. This is an important step toward the development of a clinically useful rehabilitation tool for this patient population.

## Conflict of Interest Statement

The authors declare that the research was conducted in the absence of any commercial or financial relationships that could be construed as a potential conflict of interest.

## Supplementary Material

The Supplementary Material for this article can be found online at http://www.frontiersin.org/Journal/10.3389/fneur.2014.00066/abstract
