# Supplementary material for: Artificial Balance: Restoration of the Vestibulo-Ocular Reflex in Humans with a Prototype Vestibular Neuroprosthesis
Source: Front Neurol. 2014 Apr 29;5:66. doi: 10.3389/fneur.2014.00066 (PMC4010770; doi:10.3389/fneur.2014.00066)
Supplement: Supplementary file 1 [file Presentation1.PDF]

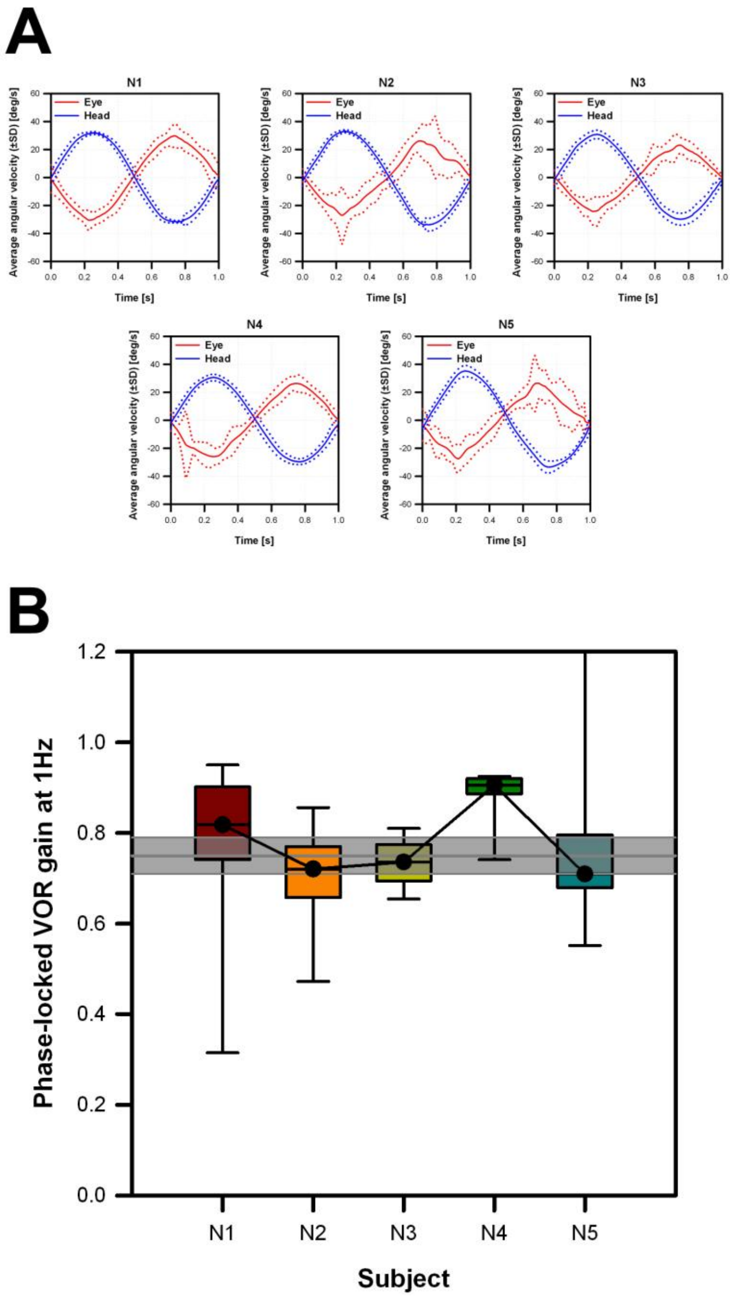

Supplementary Figure 1. VOR performance of a group of 5 healthy volunteers. Panel A (top): VOR responses of the 5 healthy volunteers to 30°/s peak-velocity sinusoidal rotations around the vertical axis in complete darkness at 1Hz. Each individual plot presents the average cycle plots (±SD) of the horizontal angular velocity of the eye (red lines) and the head (blue lines). Twenty-one cycles were analyzed in each case. Panel B (bottom): Individual phase-locked VOR gain. Data were analyzed on a cycle-by-cycle basis. Box plots indicate median values, 25th and 75th percentile values (colored box) as well as 10th and 90th percentile values (error bars). The gray bar represents the mean ±SEM of all 5 subjects.
