# Supplementary material for: Artificial Balance: Restoration of the Vestibulo-Ocular Reflex in Humans with a Prototype Vestibular Neuroprosthesis
Source: Front Neurol. 2014 Apr 29;5:66. doi: 10.3389/fneur.2014.00066 (PMC4010770; doi:10.3389/fneur.2014.00066)
Supplement: Supplementary file 2 [file Presentation2.PDF]

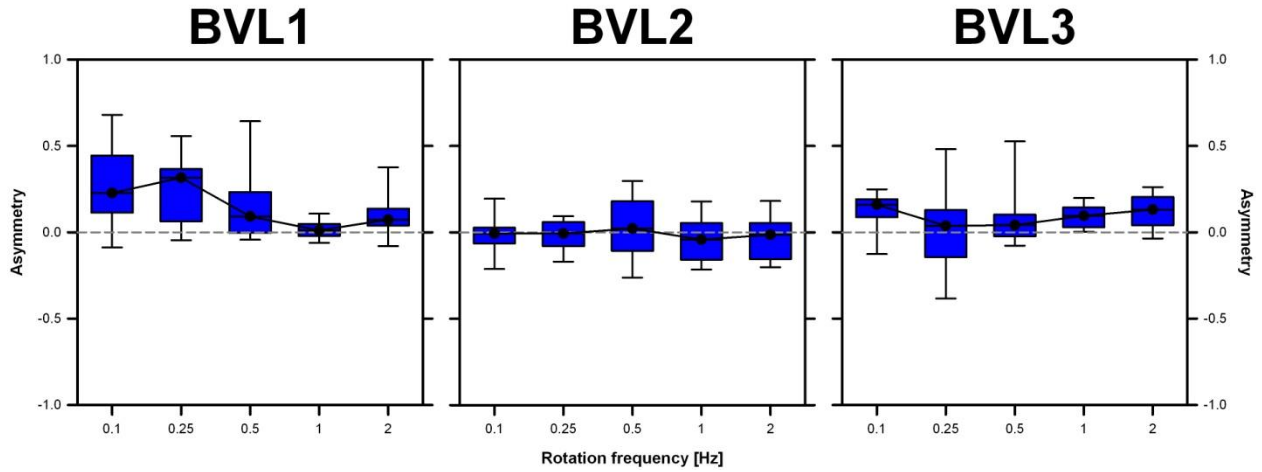

Supplementary Figure 2. Asymmetry of the electrically evoked VOR response versus rotation frequency for the three implanted patients. Asymmetry was computed as G_E-G_I/G_E+G_I, where G_E stands for the gain of the excitatory peak of the sinusoidal head movement (i.e., towards the implanted ear) and G_I stands for the gain of the inhibitory phase of the sinusoidal head movement (i.e., away from the implanted ear). G_E and G_I were obtained by calculating the best fits to stimulus half cycles (e.g., only positive or only negative; Dai et al., 2011a) . Box plots indicate median values, 25th and 75th percentile values (blue boxes) as well as 10th and 90th percentile values (error bars). At the higher rotation frequencies >0.5Hz where we effectively observed an artificial restoration of the VOR, the median asymmetry in the response was much lower than that reported in animal studies (Dai et al., 2011a, 2011b; Lewis et al., 2010), with maximums of 0.08 and 0.13 for BVL1 and BVL3 at 2Hz, and of -0.04 for BVL1 at 1Hz.
